# Resuspension and Dissemination of MS2 Virus from Flooring After Human Activities in Built Environment: Impact of Dust Particles

**DOI:** 10.3390/microorganisms12122564

**Published:** 2024-12-12

**Authors:** Stephanie A. Boone, M. Khalid Ijaz, Julie McKinney, Charles P. Gerba

**Affiliations:** 1 Department of Environmental Science, University of Arizona, Tucson, AZ 85719, USA; gerba@arizona.edu; 2 Global Research and Development for Lysol and Dettol, Reckitt Benckiser LLC, Montvale, NJ 07645, USA; khalid.ijaz@reckitt.com (M.K.I.); jmckinney.jm52@gmail.com (J.M.)

**Keywords:** viral resuspension, aerosolization, virus, fomites, dust, indoor activities

## Abstract

Resuspended particles from human activities can contribute to pathogen exposure via airborne fomite contamination in built environments. Studies investigating the dissemination of resuspended viruses are limited. The goal of this study was to explore viral dissemination after aerosolized resuspension via human activities on indoor flooring. Nylon carpet or wood flooring was seeded with virus (MS2) or virus laden dust then evaluated after activities, i.e., walking and vacuuming. Statistically significant differences were found in dispersal of virus laden dust after vacuuming carpet (*p*-value = 5.8 × 10^−6^) and wood (*p*-value = 0.003, distance > 12 in/30 cm). Significant differences were also found between floor materials and virus laden dust dispersal vacuuming (*p* = 2.09 × 10^−5^) and walking (*p* = 2.68 × 10^−2^). A quantitative microbial risk assessment (QMRA) scenario using Norovirus and a single fomite touch followed by a single hand-to-mouth touch indicated a statistically significant difference associated with virus laden dust particles and vacuuming carpet(*p* < 0.001). Infection risks were 1 to 5 log_10_ greater for dust exposure. The greatest risk reductions from fomites were seen across vacuuming carpet no-dust scenarios for surfaces <30 cm from flooring. More research is needed to determine the role resuspension plays in exposure and transmission of potentially infectious agents.

## 1. Importance 

In assessing exposure and transmission risks to infectious agents, it is important to recognize that viruses can be resuspended into the air by human activities in the indoor environment, such as walking on floors and vacuuming. Virus suspension matrix/media and/or particle size can greatly impact viral resuspension, exposure, human contact (lungs, nose, hand etc.) and transmission risk rates. The resuspended viruses may present an infection risk via in-halation or contamination of other fomites/surfaces from touching resulting in transmission via ingestion when the lips are touched. The application of quantitative microbial risk as-sessment demonstrates the potential for this risk from the contamination of fomites. 

## 2. Introduction

Interest in mechanisms of pathogen spread and interventions to control the transmission of infectious agents in the built environment has increased since the COVID-19 pandemic [1,2]. COVID-19 (caused by the virus SAR-CoV-2) resulted in 160 million cases and 3.3 million deaths worldwide in 2020, and by 2023 there were 761 million cases and 6.87 million deaths [3]. SARS-CoV-2 spread was believed to be predominately through aerosol transmission in indoor environments [3,4]. The pathway of aerosolized virus transmission is especially important for indoor settings with limited ventilation in which aerosol concentrations have built up [3]. However, the impact of aerosol resuspension and fomite transmission routes for COVID-19 or other respiratory viruses is uncertain [5]. Humans spend 90% of their time in the built environment (e.g., homes, schools, office buildings, hospitals, etc.) [6,7,8]. and exposure to microorganisms from human activity often represents a hidden hazard or unidentified risk factor [9]. The surfaces of indoor inanimate objects can be sources of microbes containing a variety of bacteria, viruses, fungi, archaea, and protistans that may include pathogens [8]. Particles that are resuspended indoors can increase the risk of microbial and pathogen exposure via ingestion and inhalation [10]. Many research studies have investigated the presence or persistence of viruses on indoor surfaces and in air [6]. Studies using polymerase chain reaction (PCR) have detected a variety of viruses on indoor surfaces/fomites, including influenza A, norovirus, picornaviruses, human rhinovirus, rotavirus, coronavirus (SARS, MERS, and COVID-19), human adenovirus, parainfluenza, and poxviridae (monkey pox and smallpox) [8,11,12]. Infectious viruses have also been found on both fomites and in the air in homes and hospitals (SAR-CoV-2 and Monkey pox) [11,12]. Humans can shed microbes directly into indoor air or onto surfaces, transport microbes from the outdoors to the indoors (on clothing, shoes, in our hair etc.), and acquire microbes from indoor surroundings (e.g., kitchen and bathroom surfaces, air) [8].

**Figure 1:**
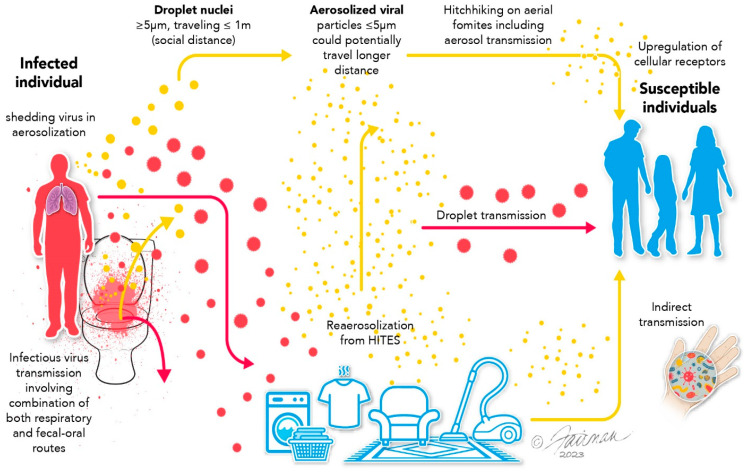
Resuspension or re-aerosolization of virus after settling on environmental surface (Floor). Modification from [11].

Virus transmission routes in indoor environments are diverse and include direct and indirect contact, fecal–oral transmission, and droplet and airborne transmission. During primary aerosolization, respiratory droplets and aerosols are released or emitted into the air during talking, breathing, coughing, or sneezing [13,14]. A cough can generate approximately 3000 droplets, or a sneeze up to 40,000 droplets [15] and each droplet may have millions of viruses released at rate of 48–320 km/h [16]. Respiratory aerosols produced by an infected person can vary between 0.3 and 2000 microns in size, and droplet (>5.0 microns) size is driven by solute content (e.g., mucus, dust, debris) [17,18]. Emission respiratory particles are deposited onto surfaces through various mechanisms including Brownian motion, gravity, thermophoresis, and electrophoresis [13,14]. Relatively large respiratory droplets (>5 µm) travel short distances (1–2 m) before settling on surfaces where viruses remain infectious from 3 h to 28 days [18,19]. Secondary aerosolization, re-aerosolization, or resuspension occurs when settled particles from the surfaces are detached or re-entrained into the surrounding air [13,14]. (Figure 1). Resuspended particles from human activities are a major source of biological and non-biological indoor aerosols [3,13]. Viruses contained within fine aerosols (small particle ≤ 5 µm) or/and dust particles can remain suspended for extended time periods and can be transported over lengthy distance by air currents [18,20]. The Baig study [21], used bovine coronavirus to evaluate ventilation system impact on virus aerosolization, deposition, and resuspension. The study found that there was a stronger virus association on plastic and metal surfaces with a higher dissociation (resuspension) from wood surfaces. PRD-1 phages were also disseminated in the room to evaluate movement, and higher amounts of virus were found above the patient’s head and near the foot of the bed. The Alhaji study [22] applied Ansys Fluent CFD simulation tools to evaluate the aerodynamic force influence on surfaces and the deposition and resuspension of coronavirus, and found that surface roughness characteristics influences the resuspension rate.

Airborne particulate matter (PM) can impact human health and is linked to cardiopulmonary and respiratory diseases [23]. Resuspended dust can constitute up to 60% of particulate matter in indoor air [24]. An early study by Thatcher and Layton [25], reported dust resuspension rate coefficients ranging from 10^−5^ to 10^−4^ when four people were walking around indoors. The Qian and Ferro [26] study assessed the effects of relative humidity, flooring type, walking, person weight, and ventilation patterns on particle concentration and reported resuspension coefficients ranging from 10^−6^ to 10^−2^ per hour. Resuspended dust has been shown to contain bacteria [27] and viruses [28]. Exposure to pathogens via floor dust has been recognized as a possibility [24,29,30]. Khare and Marr [24] theorized that people walking across a floor indoors can generate a vertical concentration gradient of resuspended dust that may lead to increased dust exposure close to the floor or/and at lower heights. Understanding exposure to dust is important and has resulted in a variety of studies on indoor dust resuspension [24].

Specific activities and movement by people in a room can result in increased dust particle concentration due to shedding and resuspension from the floor and other surfaces [24]. However, the role of resuspension remains an open research question, and more studies are needed to determine its importance in infectious agent transmission and spread [13]. A few studies have investigated the dynamics of viral transport and resuspension via microbial aerosol fallout or deposition on to environmental fomites or surfaces using dust and indoor human activities. In a study by Wu [31], a robotic infant was placed in a closed chamber and simulated crawling on a carpeted floor with dust. The study found that dust particles were resuspended during crawling with a significant amount of the particles inhaled during dust exposure. Also, dust resuspension patterns were found to be comparable to an adult walking on carpet and varied with infant weight and carpet type [31]. The Asadi [32] animal study suggests that aerosolized fomites may contribute to Influenza viral transmission. Virus-contaminated dust particles (aerosolized fomites) generated from inanimate objects were aerosolized and carried virus to a susceptible guinea pig. This study was restricted to virus transport over a very short distance or proximity, and the animal movements in no way model human activities or behaviors in the built environment. The study by Rawat [3], nebulized live Influenza A virus H3N2 in a sealed BSL-2 chamber, and virus was allowed to accumulate onto surfaces with and without inorganic dust, then viral resuspension was measured after a person walked for 20 min. Both RT-PCR results and cell culture were used to quantify viable viruses and Rawat’s [3] study, detected low levels of Influenza after 5 h.

Phage models have been compared and developed as eukaryotic virus surrogates in simulating bioaerosols and MS2 has been the most broadly used surrogate in aerosol studies [17]. The objective of this study was to explore the scope of viral dissemination and the dynamics of resuspension (e.g., vertical and horizonal distance) after performing activities on indoor flooring (carpet and wood), and to use the resulting data to estimate indoor viral exposure and disease risk using QMRA. This goal will assist with identifying possible routes of viral transmission, and human behaviors or activities that may increase or decrease the risk of viral transmission. The data from this research could be useful in assessing interventions to reduce the spread of viruses in built environments.

## 3. Materials and Methods

### 3.1. MS-2 Preparation

MS-2 bacteriophage was used as a surrogate for pathogenic human viruses. *Escherichia coli* (ATCC 15597) and bacteriophage MS-2 or *Emesvirus zinderi* (ATCC 15597-B1) were obtained from the American Type Culture Collection (ATCC, Manassas, VA, USA). MS-2 coliphage was prepared as previously described with minor modifications [33]. Briefly, 0.1 mL of phage suspension and 0.3 mL of a log-phase *E. coli* 15597 (host bacterium) culture were added to top agar, and the agar was melted and maintained at 45 °C water bath. The inoculated top agar was mixed and poured over Tryptic Soy Agar (TSA) (Difco, Franklin Lakes, NJ, USA). The solidified agar overlay plates were then inverted and incubated at 37 °C for 24 h. Tryptic Soy Broth (TSB) (Difco, Franklin Lakes, NJ, USA) was then added to each plate and maintained at room temperature for 2 h. Phosphate-buffered saline (PBS) or Tryptic Soy Broth (TSB) (MP Biomedicals, Santa Ana, CA, USA) eluates were aspirated and centrifuged to removal bacterial debris, after which the supernatants were filtered through 0.22 µm pore size Steriflip filters (Millipore Sigma, Darmstadt, Germany). The coliphage stock was stored at 4 °C until use. A tripartite solution of yeast extract, bovine albumin, and Tryptic Soy Broth (TSB) was used to mimic an organic soil load and added to 10^11^ MS2 per ml in a 20 to 15 ratio [33]. This solution was inoculated directly to the carpet or wood flooring in 50 µL droplets over a 30 × 30 cm^2^ area. A total of 10 mL phosphate-buffer solution (PBS) was added to Petri dishes as media for MS2 settle plates. The dust used in this experiment consisted of one gram of ISO 12103-1. A2 fine dust (Power Technologies Inc. (PTI), Arden Hills, MN, USA) was applied to flooring using a Mesh sieve # 400, 38 microns, 0.0015 inches S/N 201934548 to filter out larger grains of dust.

### 3.2. Experimental Conditions and Assays

All research personnel wore Tyvek suits, gloves, and N95 masks while conducting the experiment and when collecting the samples to avoid cross contamination. The experiment was replicated 3 to 5 times, and the average values were used in the data. All experiments were conducted in a 5.8 × 7.3 m room with 2.4 m ceilings. Studies suggest that ventilation rate and airflow patterns contribute directly to the spread of airborne infection [15], so to control air currents, the room was sealed with no ventilation, no windows, and one door that was sealed at the bottom, top, and around the edges. The temperature was maintained at 22.2 ± 1.8 °C with 50 ± 5% relative humidity. The flooring consists of a 0.6 × 3 m section of nylon carpet with cut medium pile or wood plank flooring, (Home Depot, Atlanta, GA, USA). The flooring was at least one meter from the room walls on all sides. The flooring was adhered to the existing flooring and a 30 × 30 cm^2^ was taped to the center of the flooring for identification of the site of inoculation. Inoculated flooring was allowed to air-dry for one hour after MS-2 inoculation. The vacuum used in all experiments was an upright Eureka Air Speed (Eureka, Parsippany, NJ, USA) without a high efficiency particulate matter (HEPA) filter. Settle plates (Petri dishes containing media) (100 mm in diameter) containing 10 mL of phosphate-buffered saline were placed at designated sample collection locations. Settle plate lids were removed after floor or carpet inoculation dried [9]. Immediately after drying, research personnel walked on flooring or vacuumed the length of the floor (4 times). Personnel immediately left the room after each activity, and generated aerosols were allowed to settle for one hour. PBS was collected from each settle plate and individually assayed and quantified as previously described [33].

Control samples were used to test the recovery efficiency of the virus from flooring. Controls consisted of a 30 × 30 cm^2^ square of flooring or carpet placed in a corner, inoculated, but no activity (vacuum or walking) took place on the surfaces. Samples of the vacuum roller, vacuum lint, and room floor were also collected to determine the spread from vacuum roller. 3M Sponge sticks (3M Corporation, St. Paull, MN, USA) and settle plates were used to collect control and vacuum samples. The room and flooring were cleaned with 1% hypochlorous acid solution after and before each experiment and tested to ensure no infectious MS-2 was present.

**Figure 2:**
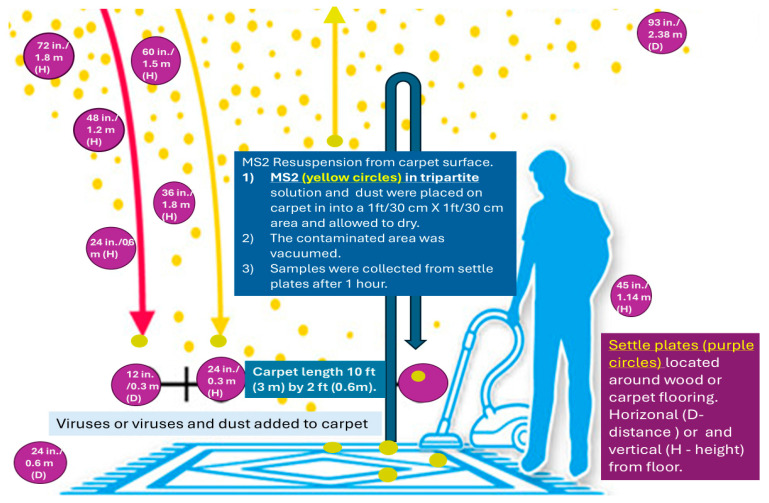
Description of experiment and positions of the MS2 settle plates during evaluation of viral spread after walking on and vacuuming indoor flooring. Purple circles are examples of settle plate horizontal distance (D) and vertical distance or heights (H) above flooring (carpet or wood).

This study evaluated horizonal (height) and vertical (distance from inoculation site) after virus dispersal/spread using settle plates (defined in lines 181 and 182). Settle plates were placed on both sides of the flooring, as seen in Figure 2.

### 3.3. Statistical Methods

#### Quantitative Microbial Risk Assessment Exposure Model

Because norovirus could not be safely suspended in the chamber, a quantitative microbial risk assessment was conducted to estimate the risks posed by norovirus resuspension from floors onto surfaces of various heights and distances. Phage concentrations could not be directly used since phages must be propagated in high concentrations for experimental detection. Due to uncertainties about the concentration of infectious viruses on floors, a wide range was explored to evaluate how risks would vary according to varying bioburden levels. Concentrations between 10^4^ and 10^8^ viral particles/cm^2^ were sampled to inform floor concentrations ($C_{floor},$ viral particles/cm^2^). Ratios of concentrations of phage on surfaces at heights vs. floors were calculated (R, unitless) to inform a factor used to estimate concentrations of norovirus in surfaces at various heights from dust or tripartite suspensions (Equation (1)):

(1) $$C_{surf}=C_{floor}R$$

The modeled exposure event was a single fomite touch followed by a single touch with the mouth to estimate infection risk. This has been adopted in other QMRAs to compare fomite-mediated risks across different scenarios or contamination levels per surface type [19]. Twenty-four scenarios were explored: 3 heights from the floor (<30 cm, 55–105 cm, or >122 cm) × 2 activities (walking, vacuuming) × 2 surface types (carpet, hardwood flooring) and from virus suspended in 2 media types (dust, tripartite). Tripartite is an organic substrate used to stimulate organic suspensions of human bodily fluids and solids (e.g., mucus) [34].

To calculate a concentration on the hands after a single fomite touch ($C_{hand}$, viral particles/cm^2^), Equation (2) was used, where $C_{surf}$ is the norovirus concentration on the surface (viral particles/cm^2^), ${TE}_{SH}$ is the surface-to-hand transfer efficiency (fraction, unitless), and $S_{H}$ is the fraction of the hand used for the contact (fraction, unitless).

(2) $$C_{hand}=C_{surf}{TE}_{SH}S_{H}$$

Transfer efficiencies from surface to finger pad for MS2 on stainless steel surfaces reported by [35], were used to inform the mean and standard deviation of a normal distribution (range 0–1) that was randomly sampled for ${TE}_{SH}$. The fraction of the hand used for a contact was informed by [35], using fractions measured for adults using a front partial fingers or a full front palm configuration. The front partial fingers were divided by 5 to capture a single fingertip, so that configurations as small as a single fingertip up to a full front palm with fingers were accounted for. The minimum and maximum values for these configurations and adjustments, in the case of the front partial fingers configuration, were used to inform the minimum and maximum of a uniform distribution.

A dose (number of viral particles) was then calculated using Equation (3), accounting for virus that transfers to the mouth for a single hand-to-mouth contact, where ${TE}_{HM}$ is hand-to-mouth transfer efficiency, $S_{M}$ is the fraction of hand surface area used for the hand-to-mouth contact, and $A_{hand}$ is the surface area (cm^2^) for a single hand. A normal distribution for transfer efficiencies for hand-to-mouth contacts (range 0–1) was informed by the mean and standard deviation reported by [34], using finger-to-lip transfer efficiencies for ASTM tripartite soil load. Total hand surface area for a single hand was informed by a distribution used by [36], informed by the U.S. Environmental Protection Agency Exposure Factors Handbook [37].

(3) $$Dose=C_{hand}{TE}_{HM}S_{M}A_{hand}$$

Doses were then inputted into a fractional Poisson dose–response equation informed by Van Abel [38]. This dose–response curve (Equation (4)) assumes aggregation and is from a fit of pooled dose–response data for GI (8fIIa + b) and GII.4 [38]. The curve for aggregated virus was used because viruses tend to aggregate upon drying on surfaces [39]. While there is debate about which curves best represent norovirus dose–response across various scenarios [38], the absolute infection risk estimates in this study were not the focus. Rather, determining percent changes in infection risk for tripartite relative to dust scenarios to estimate approximate risk reduction benefits in reducing dust burdens in indoor environments was the aim. More data are needed to determine which curve would be the most appropriate for dust and non-dust fomite-mediated norovirus transmission in indoor environments.

(4) $$P_{risk}=P\cdot \left(1-e^{\frac{-Dose}{u_{a}}}\right)$$

Infection risks were compared between dust and tripartite for reach height and activity scenario. All model parameters can be found in Table 1.

If we consider the “tripartite” scenario to represent an intervention in which dust is greatly reduced as a potential vehicle for viral dispersion from floors, a log_10_ reduction in norovirus infection risk can be calculated assuming an intervention of dust removal before vacuuming or walking activities on carpet or hard floors. This approach was taken, and log_10_ reductions in risk (log_10_ ($P_{infection,dust}/P_{infection,tripartite})$) were calculated.

## 4. Results

### 4.1. Experimental Outcome

**Figure 3:**
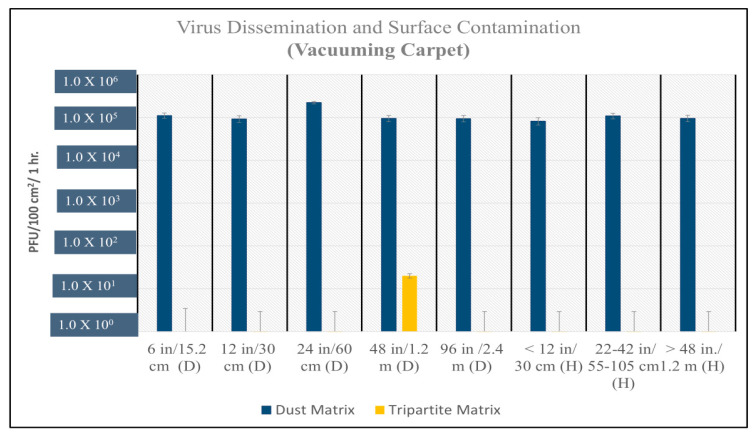
Average resuspension and surface contamination of MS2 bacteriophage (PFU) after vacuuming carpet with dust matrix on floor (blue bars) and tripartite matrix (yellow bars). Statically significant difference found between dust matrix and tripartite matrix (*p*-value = 5.8 × 10^−6^). H is height of settle plate from ground; all vertical settle plates were less than 0.6096 m from initial contamination site; (D) is distance of settle plate from initial contamination site on carpet. Many samples taken from carpet with no dust were determined to be below the limit of detection (LOD or 5 [5.0 × 10^1^]).

**Figure 4:**
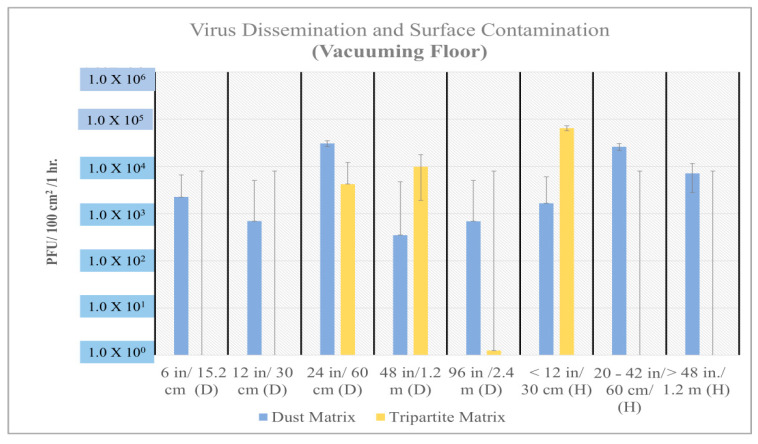
Average resuspension of MS2 after vacuuming wood flooring. Statistically significant (*p* value = 0.003) for viral spread if distance is <12 inches, if dust is present.

**Figure 5:**
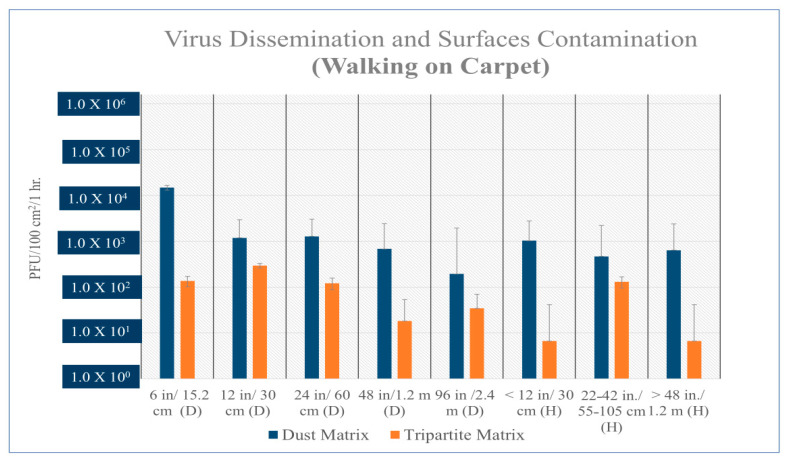
Average resuspension of MS2 after walking on carpet.

**Figure 6:**
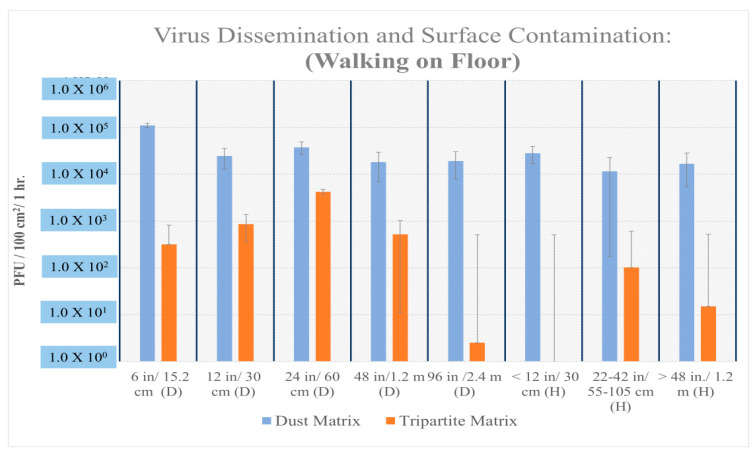
Average spread and aerosol surface contamination of MS2 after walking on wood flooring.

The recovery of viable MS2 virus from vertical settled plates ranged from 10^5^ to 10^6^ PFUs (plaque-forming units/100 cm^2^/h) after vacuuming virus and dust laden (dust matrix) contaminated carpet. The recovery of viable viruses was below the limit of detection when the carpet contained no added dust (tripartite matrix) (Figure 3, Figure 4, Figure 5 and Figure 6). A two-tailed T test was used to evaluate statistical differences between experimental conditions. The virus spread (both distance and height) was statistically significant, *p*-value = 5.8 × 10^−6^ when virus was associated with dust and the carpet was vacuumed (Figure 3). Viral dissemination was also statistically significant if dust was present when vacuuming wood flooring but only at short distances ≤ 12 inches (*p*-value = 0.003) and heights (vertical transport) > 20 inches (*p*-value = 0.05). 

If virus laden dust was present on the flooring a statistically significant difference was found between floor materials and virus dissemination or spread during vacuuming (*p* = 2.09 × 10^−5^) and walking (*p* = 2.68 × 10^−2^). Generally, if wood floor was contaminated with dust, viable viruses were recovered from both vertical and horizonal settle plates and both contained a higher quantity of virus as compared to flooring with no dust. Samples without dust were random and lower in viral concentration, and the majority of viruses were recovered in the front and back of the vacuum cleaner when it was at standstill. Further testing indicated viable viruses in vacuum lint samples (average 5.95 × 10^5^), and in carpet samples from the front (4.69 × 10^4^) and back (1.37 × 10^5^) of the vacuum cleaner when at a standstill. The vacuum cleaner roller also contained an average 4.20 × 10^7^ virus. The resulting dispersal of virus on settle plates after vacuuming is consistent with the steady rhythmic movement of the vacuum brush against the carpet or wood floor. Both experiments reflected the significant increase in virus dispersal when dust matrix was present, and the vacuum was used. When walking on the carpet, no statistically significant difference was found between virus dispersal; however, viral dissemination was consistently higher if dust was present (Figure 5). 

### 4.2. Quantitative Microbial Risk Assessment Results

The ratios of concentrations of phage on surfaces compared to on floors, used to inform concentrations in the QMRA, are visually represented in Figure S1 and Table S1. The concentrations on floors ranged from 6.0 × 10^8^ PFU/100 cm^2^ to 1.4 × 10^10^ PFU/100 cm^2^. Larger fractions of virus on surfaces to virus on floors were seen for dust experimental trials relative to tripartite, except for <30 cm surfaces on hard flooring following vacuuming (Figure S1, Table S1). There were no consistent patterns in these ratios across heights of surfaces from the floor (Figure S1, Table S1).

**Figure 7:**
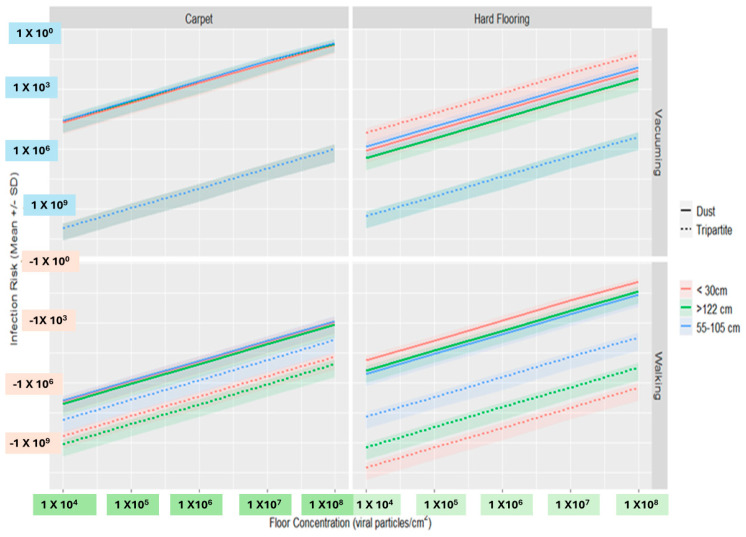
Mean ± SD of norovirus infection risks for a single touch with a surface <30 cm, 55–105 cm, or >122 cm from the floor following walking and vacuuming on carpet or hard flooring, exploring a wide floor viral bioburden range.

Estimated norovirus infection risks from contacts with surfaces given a wide range of viral concentrations on floors were consistently higher for dust scenarios relative to tripartite scenarios for both vacuuming and walking on carpet and walking on hard flooring. In the case of vacuuming on hard flooring, norovirus infection risks were greater for surfaces closer to the floor under tripartite scenarios than for surfaces at all distances from the floor for dust scenarios (Figure 7).

Estimated log_10_ reductions in norovirus infection risk from single fomite touches due to the removal of dust before vacuuming or walking on carpet or hardwood flooring ranged from 1.0 to 5.4, except for surfaces less than 30 cm from the floor following vacuuming on hard flooring. This was because higher virus concentrations were observed in the tripartite trials for this scenario relative to the dust trials. The greatest risk reductions were estimated for single touches on surfaces >55 cm for the vacuuming on carpet scenario and for surfaces <30 cm from the floor for the walking on hard flooring scenario. This indicates that dust could contribute higher viral loads on surfaces at these heights following these activities and that the removal of dust could reduce fomite-mediated risks originating from touches with these surfaces followed by these activities, specifically. These log_10_ reductions in infection risk are similar to the log_10_ differences in measured viral concentrations themselves (Table 2), which is expected due to the proportional relationship between viral concentration and dose in the utilized QMRA model and the linear log_10_ relationship of dose with log_10_ infection risk.

## 5. Discussion/Summary

Studies by Rawat [3], quantified resuspended viral particles after 5 h (not cumulative), and investigated respiratory virus (Influenza) transport via dust laden particles after walking. The 5 h samples collected after resuspension would have consisted of <1 μm particles or those particles pertaining to aerosol transport [11]. This study investigated the dissemination of aerosol particles >2.0 μm that would have fallen to surfaces over a one-hour period, those larger particles would be included in exposure to fomites, and these larger droplets may contain large amounts of virus or aggregates of virus.

In this study, we used a single-stranded RNA virus/bacteriophage which is similar in structure and composition to Enteroviruses, and rhinoviruses. Bacteriophage MS2 has been used previously to study the dispersion of viruses in the built environment [36,41]. Additionally, settle plates were used to assess MS2 bacteriophage fallout or the dissemination of viral aerosols resulting from virus being resuspended by activities conducted on indoor flooring. In the presence and absence of dust, this study measured the fallout from viral resuspension at various heights above the floor and various distances from the point of viral inoculation. The advantage of using bacteriophage is that no cell culture is needed to grow the virus; cell culture results can vary or give differing results depending on the cell line used. Cell culture is also time-consuming, expensive, and can be susceptible to toxic chemicals and bacterial infections, making false positives a possibility. Since bacteriophage can be used as a surrogate for enterovirus and respiratory viruses (single-strand RNA virus) without the variation of cell culture, the procedure is deemed to be an accurate assessment of viral viability.

Viral viability in the air and on indoor surfaces can be influenced or affected by relative humidity, temperature, sunlight, viral characteristics, and the surrounding matrix [42]. Research indicates that particle generation and particle size or aerosolization are key determinates for pathogen carriage, aerosolization, transmission, and exposure [42]. Small particles (<5 μm) can stay suspended for prolonged periods and are associated with airborne pathogen transmission [42]. Larger particles (>5 μm) settle on surfaces or the ground relatively quickly and are associated with droplet and fomite transmission [42]. Our study investigated the spread and resuspension fallout of large particles facilitated by human activity on indoor flooring, or particles that may result in droplet or fomite transmission.

This study’s Quantitative Microbial Risk Assessment (QMRA) model focused on the virus dissemination or spread after walking on and vacuuming a floor surface (carpet or hardwood), modeling virus spread by the fecal oral route and not by inhalation. Vacuuming of a floor was believed to play a role in outbreaks in hotels in which areas of carpeting were vacuumed following vomit contamination by an ill person [43,44]. It has also been suggested that airborne spread is possible from inhalation and subsequent ingestion of virus particles [45].

To assess the potential risk of infection after resuspended particles settled onto surfaces, a QMRA approach was used to simulate the risk of infection from a norovirus-contaminated floor from touching a surface contaminated by the resuspended virus. Infection risks were roughly 1 to 5 log_10_ greater for dust relative to tripartite scenarios, except for surfaces <30 cm from the floor following vacuuming on hard flooring. The greatest reductions in risk from fomites for lower dust conditions were seen across vacuuming on carpet scenarios and for surfaces <30 cm from the floor following walking on hard flooring, except for surfaces <30 cm from the floor following vacuuming on hard flooring. The greatest reductions in risk from fomites for lower dust conditions were seen across vacuuming on carpet scenarios and for surfaces <30 cm from the floor following walking on hard flooring. Diverse and disease-causing microorganisms and pathogens are typically carried though aerosols [46]. Aerosolized resuspended particles from indoor activities are known to play a large role in human exposure to many pollutants [13]. Surface contact is a common pathway for the infection to spread among people within the indoor built environment [21]. The activities simulated in this study demonstrated that the virus was resuspended to heights within the inhalation range of both children and adults. This study indicated that typically, more viruses were dispersed both vertically and horizontally if dust was present during walking and vacuuming. It has also been suggested that airborne spread of norovirus is possible from inhalation and subsequent ingestion of virus particles [45]. Results also suggest that resuspension offers the potential of respiratory virus transmission by the resuspension of previously settled virus on flooring. The risks from resuspension may be greater for children who crawl and are more likely to play on the floor. Increased exposure may also occur at lower heights, which may include small children and other household activities (i.e., sitting on a couch or at a desk). Children are a part of the vulnerable population and are facing rising threats from infectious disease [47]. Environmental factors impact infectious disease, and reports highlight the close association between emerging infectious disease and environmental factors [48]. Our study data suggests that children can be negatively impacted by viral diseases because of the increased risks associated with resuspension and deposition of virus onto fomites or/and an increased frequency of exposure to contaminated indoor air and surfaces. Thus, regular disinfecting of surfaces could be beneficial in the dissemination of viruses spread by both ingestion and inhalation.

This study indicates that more research is needed to evaluate the relative contribution of virus-laden dust on infection risk in indoor environments and explore the potential for air quality and surface hygiene interventions to reduce risks posed by resuspended particles.

## Figures and Tables

**Table 1:** Model parameters, distributions, and sources.

Parameter		Variable	Units	Point Value or Distribution	Source
Transfer efficiency	Surface-to-hand	${TE}_{SH}$	Fraction (unitless)	Normal (mean = 0.34, SD = 0.12), range 0–1	[35]
	Hand-to-mouth	${TE}_{HM}$	Fraction (unitless)	Normal (mean = 0.41, SD = 0.1098), range 0–1	[34]
Fraction of the hand	Surface contacts	$S_{H}$	Fraction (unitless)	Uniform (min = 0.008, max = 0.25)	[40]
	Mouth contacts	$S_{M}$	Fraction (unitless)	Uniform (min = 0.008, max = 0.012)	[40]
Hand surface area for a single hand		$A_{hand}$	cm^2^	Uniform (min = 445, max = 535)	[36,37]
Norovirus concentration on floors		Log_10_ $C_{floor}$	Log_10_ viral particles/cm^2^	4, 5, 6, 7, 8	This study (Assumed to explore wide range)
Ratio of concentrations on surfaces to floors		R	Fraction (unitless)	See Table S1	This study
Dose–response parameters		P	0.72	“Fraction of secretor positive (Se+) individuals who are fully susceptible”	[38]
		$u_{a}$	1106	Mena aggregate size	[38]

**Table 2:** Log_10_ mean (SD) reduction in norovirus infection risk for tripartite scenarios relative to dust scenarios.

Height Above Floor	Carpet		Hard Flooring	
	Vacuuming	Walking	Vacuuming	Walking
<30 cm	5.3 (0.04)	1.8 (0.0002)	−0.85 (0.01) *	5.4 (0.02)
55–105 cm	5.4 (0.05)	1.0 (0.0002)	3.5 (0.002)	2.2 (0.004)
>122 cm	5.4 (0.04)	2.0 (0.0001)	2.9 (0.0005)	3.8 (0.006)

* Percent changes are negative for vacuuming, hard flooring, <30 cm scenario, because the ratio of virus from the floor on surfaces at this height for tripartite scenarios was greater than for dust scenarios, resulting in a non-positive reduction in risk for dust removal.

## Data Availability

The original contributions presented in the study are included in the article/Supplementary Material, further inquiries can be directed to the corresponding authors.

## Supplementary Materials

The following supporting information can be downloaded at: https://www.mdpi.com/article/10.3390/microorganisms12122564/s1, Figure S1. Mean ± SD of fractions calculated based on measured phage concentration on surfaces (plaque-forming units (PFUs)/100 cm^2^) at various heights (<30 cm, 55-105 cm, >122 cm) after walking or vacuuming on carpet or hard flooring divided by the amount of virus seeded on the floor (PFU/100 cm^2^) for dust and tripartite seeding media. Table S1. Fraction of floor concentration on surfaces.

## References

[1] Jones B., Sharpe P., Iddon C., Hathway E.A., Noakes C.J., Fitzgerald S. (2021). Modelling uncertainty in the relative risk of exposure to the SARS-CoV-2 virus by airborne aerosol transmission in well mixed indoor air. Build. Environ..

[2] Onakpoya I.J., Henegha C.J., Spencer E.A., Brassey J., Rosca E.C., Maltoni S., Pluddemann A. (2022). Evans. D.H., Conly, J.M., and Jefferson, T. Viral cultures for assessing fomite transmission of SAR-CoV-2; a systemic review and meta-analysis. J. Hosp. Infect..

[3] Rawat A.D., Brown M.S., Roberts D.M., Ferro A.R. (2023). Resuspension of seeded particles containing live Influenza A virus in a full-scale laboratory. Buildings.

[4] Nastasi n Renninger N., Bope A., Cocran S.J., Greaves J., Haines S.R., Balasubrahmaniam N., Sturat K., Panescu J., Bibby K., Hull N.M. (2021). Persistence of viable MS2 and Phi6 bacteriophages on carpet and dust. Indoor Air.

[5] Short K.R., Cowling B.J. (2023). Assessing the potential for fomite transmission of SARS-CoV-2. Lancet Microbe.

[6] Rosario K., Fierer N., Mulle S., Luongo J., Breitbart M. (2018). Diversity of DNA and RNA viruses in indoor air as assessed via metagenomics sequencing. Environ. Sci. Technol..

[7] Klepeis N.E., Nelson W.C., Ott W.R., Robinson J.P., Tsang A.M., Switzer P., Behar J.V., Hern S.C., Engelmann W.H. (2001). Environmental Protectional Agency “The National Human Activity Pattern Survey (NHAPS): AResource for Assessing Exposure to Environmental Pollutants. J. Expo. Anal. Environ. Epidemiol..

[8] Stephens B., Azimi P., Thoemmes M.S., Heidarinejad M., Allen J.G., Gilbert A.G. (2019). Microbial exchange via fomites and implications for human health. Biol. Pollut..

[9] Pasquarella C., Pitzurra O., Savino A. (2000). The index of microbial air contamination. J. Hosp. Contam..

[10] Qian J., Peccia J., Ferro A.R. (2014). Walking induced particle suspension in indoor environments. Atmos. Environ..

[11] Ijaz M.K., Sattar S.A., Nims R.W., Boone S.A., McKinney J., Gerba C.P. (2023). Environmental dissemination of respiratory viruses; dynamic interdependencies of respiratory droplets, aerosols, aerial particulates, environmental surfaces, and contribution of viral re-aerosolization. PeerJ.

[12] Morgan C.N., Whitehill F., Doty J.B., Schulte J., Matheny A., Stringer J., Delaney L.J., Esparza R., Rao A.K., McCollum A.M. (2022). Environmental persistence of Monkeypox virus on surfaces in households of person with travel-associated infection, Dallas, Texas, USA, 2021. Emerg. Infect. Dis..

[13] Rawat A.D., Ferro A.R. (2022). Respiratory virus deposition and resuspension from indoor surfaces. Studies to Combat COVID-19 Using Science and Engineering.

[14] Ferro A.R. (2022). Resuspension. Handbook of Indoor Air Quality.

[15] Wei J., Li Y. (2016). Airborne spread of infectious agents in the indoor environment. Am. J. Infect. Control.

[16] La Rosa G., Fratini M., Libera S.D., Iaconellis M., Muscillo M. (2013). Viral infections acquired indoors through contact transmission. Ann. Ist. Super Sanita.

[17] Duchaine C. (2016). Assessing microbial decontamination of indoor air with particular focus on human pathogenic viruses. Am. J. Infect. Control.

[18] Kohanski M.A., Lo L.J., Waring M.S. (2020). Review of indoor aerosol generation, transport, and control in the context of COVID-19. Int. Forum Allergy Rhinol..

[19] Pitol A.K., Julian T.R. (2021). Community transmission of SARS-CoV-2 by surfaces: Risks and risk reduction strategies. Environ. Sci. Technol. Lett..

[20] Ijaz M.K., Zargar B., Wright K.E., Rubino J.R., Sattar S.A. (2016). Generic aspects of the airborne spread of human pathogens indoors and emerging air decontamination technologies. Am. J. Infect. Control.

[21] Baig T.A., Zhang M., Smith B.L., King M.D. (2022). Environmental effects on viable virus transport and resuspension in ventilation airflow. Viruses.

[22] Alhaji M.M. The impacts of surface roughness on indoor aerodynamics of virus laden particles: The case of contact, deposition and resuspension. Proceedings of the 11th International Conference for Indoor Air Quality, Ventilation and Energy Conservation in Buildings.

[23] Middleton J., Lopes H., Michealson K., Reid J. (2020). Planning the second wave pandemic of COVID-19 and planning for winter: A statement from the association of scholls of public health in the European region. Int. J. Public Health.

[24] Khare P., Marr L.C. (2014). Simulation of vertical concentration gradient of influenza viruses in dust resuspended by walking. Indoor Air.

[25] Thatcher T.L., Layton D.W. (1995). Deposition, resuspension, and penetration of particles within residence. Atmos. Environ..

[26] Qian J., Ferro A.R. (2008). Resuspension of dust particles in a chamber and associated environmental factors. Aerosol Sci. Technol..

[27] Hospodsky D., Qian J., Nazaroff W.W., Yamamoto N., Bibby K., Rismani-Yazdi H., Peccis J. (2012). Human occupancy as a source of indoor airborne bacteria. PLoS ONE.

[28] Evans M.R., Meldrum R., Lane W., Gardner D., Ribeiro C.D., Gallimore C.I., Westmoreland D. (2002). An outbreak of viral gastroenteritis following environmental contamination at a concert hall. Epidemiol Infect..

[29] Mohanty S.K., Leela K.S. (2013). Textbook of Immunology.

[30] Black J.G. (2012). Microbiology: Principles and Exploration.

[31] Wu T., Fu M., Martin V., Taubel M., Xu Y., Boor B.E. (2021). Particle resuspension dynamics in the infant near-floor microenvironment. Environ. Sci. Technol..

[32] Asadi S., Bouvier N., Wexler A.S., Ristenpart W.D. (2020). The coronavirus pandemic and aerosols: Does COVID-19 transmit via expiratory particles?. Aerosol Sci. Technol..

[33] Gerba C. (2000). Indicator Microorganisms. Environmental Microbiology.

[34] Abney S.E., Wilson A.M., Ijaz M.K., McKinney J., Reynolds K.A., Gerba C.P. (2022). Minding the matrix: The importance of inoculum suspensions on finger transfer efficiency of virus. J. Appl. Microbiol..

[35] Anderson C.E., Boehm A.B. (2021). Transfer rate of enveloped and nonenveloped viruses between fingernails and surfaces. Appl. Environ. Microbiol..

[36] Beamer P., Plotkin K.R., Gerba C.P., Sifuentes L.Y., Koenig K.W., Reynolds K.A. (2015). Modeling of human viruses on hands and risk of infection in an office workplace using micro-activity data. J. Occup. Environ. Hyg..

[37] U.S. EPA (2011). Exposure Factors Handbook.

[38] Van Abel M., Schoen M.E., Kissel J.C., Meschke J.C. (2017). Comparison of Risk Predicted by Multiple Norovirus Dose-Response Models and Implications for Quantitative Microbial Risk Assessment. Risk Anal..

[39] Gerba C.P., Bentancourt W.Q. (2017). Viral aggregation: Impact on virus behavior in the Environment. Environ. Sci. Technol..

[40] Auyeung W., Canales R.A., Leckie J.O. (2008). The fraction of total hand surface area involved in young children’s outdoor hand-to-object contacts. Environ. Res..

[41] Sifuentes L.Y., Kornig D.W., Phillips R.L., Reynolds K.A., Gerba C.P. (2023). Use of Hygiene Protocols to Control the Spread of Viruses in a Hotel. Food Environ. Virol..

[42] Galton J., Tovey E., Mc laws M., Rawlinson W.D. (2011). The role of particle size in aerosolized pathogen transmission: A review. J. Infect..

[43] Cheesbrough J.S., Green J., Gallimore C.I., Wright P.A., Brown D.W.G. (2000). Widespread environmental contamination with Norwalk-like viruses (NLV) detected in a prolonged hotel outbreak of gastroenteritis. Epidemiol. Infect..

[44] Kimura H., Nagano K., Kimura N., Shimizu M., Ueno Y., Morikane K., Okabe N. (2011). A norovirus outbreak associated with environmental contamination at a hotel. Epidemiol. Infect..

[45] Marks P.J., Vipond I.B., Carlisle D., Deakin D., Fey R.E., Caul E.O. (2000). Evidence for airborne transmission of Norwalk-like virus (NLV) in a hotel restaurant. Epidemiol. Infect..

[46] Atta H.I. (2023). Aerosolization and bioaerosols. Aeromicrobiology: Developments in Applied Microbiology and Biotechnology.

[47] Lou Y., Lou X., Du M., Chen Z., Long L. (2024). Editorial: Prevalent disease in vulnerable populations: Current situations ans influencing factors. Front. Public Health.

[48] Liu Q., Deng J., Yan W., Qin C., Diu M., Wang Y., Zhang S., Lui M., Liu J. (2024). Burden and trends of infectious disease mortality attributed to air pollution, unsafe water, sanitation, and hygiene, and non-optimal temperature globally and in different socio-demographic index regions. Glob. Health Res. Policy.

