# Supplementary material for: Resuspension and Dissemination of MS2 Virus from Flooring After Human Activities in Built Environment: Impact of Dust Particles
Source: Microorganisms. 2024 Dec 12;12(12):2564. doi: 10.3390/microorganisms12122564 (PMC11678224; doi:10.3390/microorganisms12122564)
Supplement: Supplementary file 1 [file microorganisms-12-02564-s001.zip › microorganisms-3298759-supplementary.pdf]

Supplemental Materials

Table S1. Fraction of floor concentration on surfaces

Floor type	Activity	Virus Application	Height	Mean ± SD
Carpet	Vacuuming	Dust	< 30 cm	3.7×10^{-5} (2.1×10^{-5})
Carpet	Vacuuming	Tripartite	< 30 cm	1.9×10^{-10} (1.6×10^{-10})
Carpet	Walking	Dust	< 30cm	2.0×10^{-7} (2.7×10^{-7})
Carpet	Walking	Tripartite	< 30cm	3.6×10^{-9} (6.0×10^{-9})
Hard Flooring	Walking	Tripartite	< 30 cm	9.7×10^{-11} (4.4×10^{-12})
Hard Flooring	Walking	Dust	< 30 cm	2.2×10^{-5} (2.9×10^{-5})
Hard Flooring	Vacuuming	Tripartite	< 30 cm	1.1×10^{-5} (1.9×10^{-5})
Hard Flooring	Vacuuming	Dust	< 30 cm	1.5×10^{-6} (2.1×10^{-6})
Carpet	Vacuuming	Dust	55 – 105 cm	4.6×10^{-5} (1.9×10^{-5})
Carpet	Vacuuming	Tripartite	55 – 105 cm	1.9×10^{-10} (1.6×10^{-10})
Carpet	Walking	Dust	55 – 105 cm	2.2×10^{-7} (1.2×10^{-7})
Carpet	Walking	Tripartite	55 – 105 cm	2.4×10^{-8} (2.9×10^{-8})
Hard Flooring	Walking	Tripartite	55 – 105 cm	3.3×10^{-8} (3.0×10^{-8})
Hard Flooring	Walking	Dust	55 – 105 cm	4.8×10^{-6} (9.2×10^{-6})
Hard Flooring	Vacuuming	Tripartite	55 – 105 cm	7.4×10^{-10} (8.6×10^{-11})
Hard Flooring	Vacuuming	Dust	55 – 105 cm	2.3×10^{-6} (3.3×10^{-6})
Carpet	Vacuuming	Dust	> 122 cm	4.5×10^{-5} (2.5×10^{-5})
Carpet	Vacuuming	Tripartite	> 122 cm	1.9×10^{-10} (1.6×10^{-10})
Carpet	Walking	Dust	> 122 cm	1.5×10^{-7} (1.6×10^{-7})
Carpet	Walking	Tripartite	> 122 cm	1.4×10^{-9} (2.4×10^{-9})
Hard Flooring	Walking	Tripartite	> 122 cm	1.0×10^{-9} (1.6×10^{-9})
Hard Flooring	Walking	Dust	> 122 cm	7.0×10^{-6} (1.4×10^{-5})
Hard Flooring	Vacuuming	Tripartite	> 122 cm	7.4×10^{-10} (8.6×10^{-11})
Hard Flooring	Vacuuming	Dust	> 122 cm	6.0×10^{-7} (7.3×10^{-7})

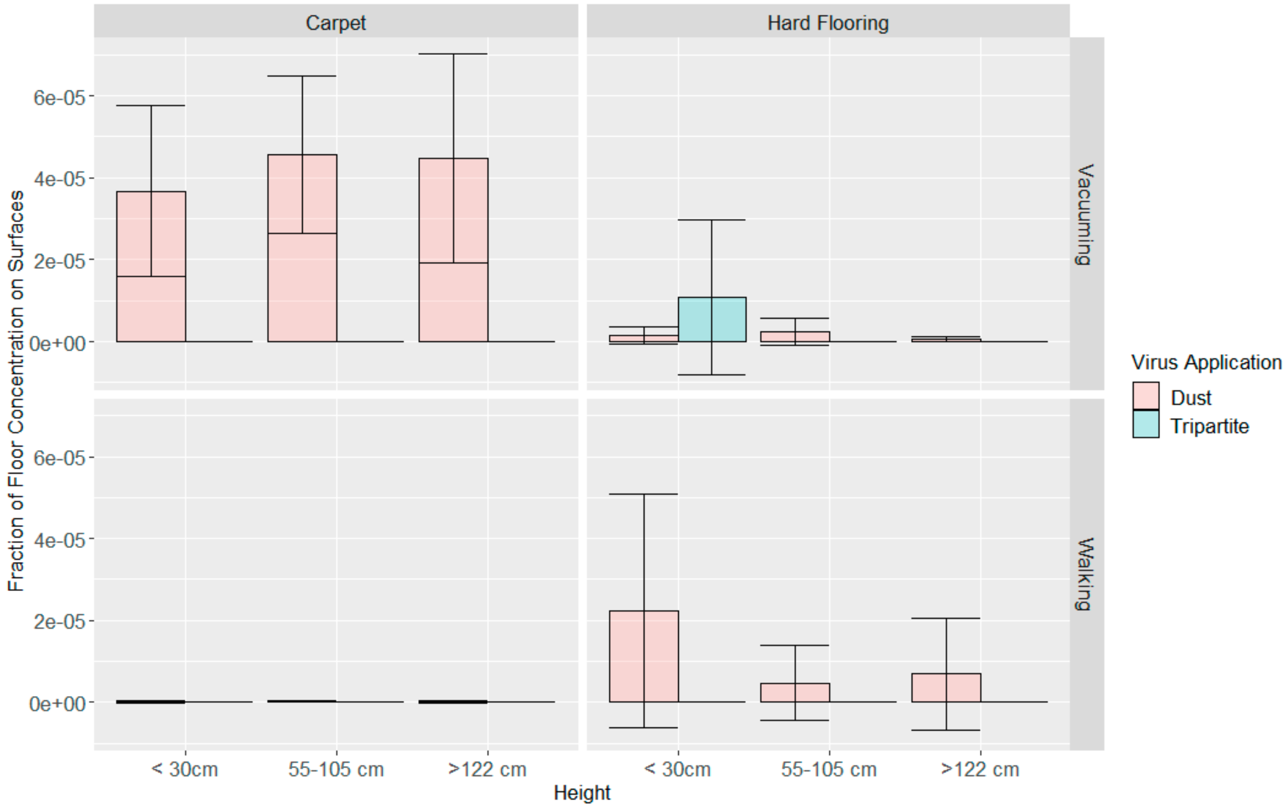

Figure S1. Mean ± SD of fractions calculated based on measured phage concentration on surfaces (plaque forming units (PFU)/100 cm^2) at various heights (<30 cm, 55-105 cm, >122 cm) after walking or vacuuming on carpet or hard flooring divided by the amount of virus seeded on the floor (PFU/100 cm^2) for dust and tripartite seeding media.
